# Motor Recovery of the Affected Hand in Subacute Stroke Correlates with Changes of Contralesional Cortical Hand Motor Representation

**DOI:** 10.1155/2017/6171903

**Published:** 2017-02-14

**Authors:** Jitka Veldema, Kathrin Bösl, Dennis Alexander Nowak

**Affiliations:** ^1^HELIOS Klinik Kipfenberg, Kipfenberg, Germany; ^2^Department of Neurology, University Hospital, Philipps-University, Marburg, Germany

## Abstract

*Objective*. To investigate the relationship between changes of cortical hand motor representation and motor recovery of the affected hand in subacute stroke.* Methods*. 17 patients with motor impairment of the affected hand were enrolled in an in-patient neurological rehabilitation program. Hand motor function tests (Wolf Motor Function Test, Action Research Arm Test) and neurophysiological evaluations (resting motor threshold, motor evoked potentials, motor map area size, motor map area volume, and motor map area location) were obtained from both hands and hemispheres at baseline and two, four, and six weeks of in-patient rehabilitation.* Results*. There was a wide spectrum of hand motor impairment at baseline and hand motor recovery over time. Hand motor function and recovery correlated significantly with (i) reduction of cortical excitability, (ii) reduction in size and volume of cortical hand motor representation, and (iii) a medial and anterior shift of the center of gravity of cortical hand motor representation within the contralesional hemisphere.* Conclusion*. Recovery of motor function of the affected hand after stroke is accompanied by definite changes in excitability, size, volume, and location of hand motor representation over the contralesional primary motor cortex. These measures may serve as surrogate markers for the outcome of hand motor rehabilitation after stroke.

## 1. Introduction

Stroke is the number one cause of long-term disability in adults worldwide [1]. About 70% of stroke survivors suffer from impaired hand motor function six months after the cerebrovascular incident even after participation in rehabilitative training programs [2]. Motor recovery of the impaired upper limb is accompanied by profound functional reorganization within motor areas of both hemispheres [3–6]. A better understanding of these processes, which may represent either “beneficial” or “detrimental” brain plasticity, may help to develop more targeted and efficient therapy strategies to overcome stroke-afflicted deficits. Over the past two decades several studies have described the changes in neural activation and cortical excitability within motor areas of both hemispheres after stroke and also their potential relationships to motor impairment and motor recovery [6]. Nevertheless, the role of the upcoming changes in cortical hand motor excitability and representation for motor recovery after stroke is still far from being completely understood. Transcranial magnetic stimulation is a sensitive tool to evaluate cortical excitability as well as size and location of cortical hand motor representation [7–12]. Here we assessed clinical measures of hand function in subacute stroke survivors and correlated these measures with measures of motor cortex excitability, cortical hand motor representation, cortical silent period, and ipsilateral silent period over a six-week period of in-patient rehabilitation.

## 2. Methods

### 2.1. Subjects

17 acute and subacute stroke patients undergoing a six-week in-patient neurological rehabilitation treatment for unilateral hemiplegia after a first unilateral stroke were included.  Table 1 presents the clinical data of all patients at baseline. Inclusion criteria were (1) location of the lesion within the territory of the MCA, (2) severe to mild sensory-motor deficit of the affected upper limb, and (3) stroke within two weeks to three months prior to study inclusion. Exclusion criteria were (1) global aphasia or cognitive impairments, which might interfere with understanding the instruction for motor testing, (2) severe neglect, (3) coexistent neurological or psychiatric illness, and (4) accepted contraindications for TMS [12]. All patients received a total of 300 daily minutes of rehabilitative training (including physiotherapy, occupational therapy, speech, and language therapy) each working day over the entire six-week period. The therapeutic sessions were centrally distributed and counterbalanced across patients.

### 2.2. Study Design

**Figure 1:**
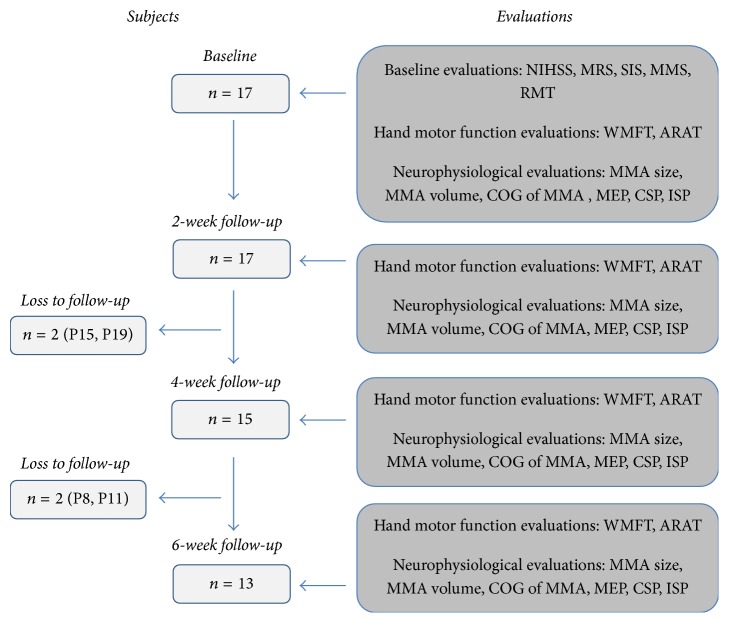
Study design. ARAT = Action Research Arm Test; COG = center of gravity; CSP = cortical silent period; ISP = ipsilateral silent period; MEP = motor evoked potential; MMA = motor map area; MMS = Mini Mental Status Examination Score; MRS = Modified Rankin Scale; NIHSS = National Institute of Health Stroke Scale; RMT = resting motor threshold; SIS = Sensibility Impairment Score; WMFT = Wolf Motor Function Test.

This prospective longitudinal study evaluates the changes of motor function of the affected and the nonaffected hands as well as the changes of neurophysiology of the affected and nonaffected hemispheres during a six-week period in stroke patients. The evaluations were performed at the baseline, two weeks, four weeks, and six weeks after study inclusion (see Figure 1). The study was approved by the Ethics committee of the Bavarian chamber of physicians.

### 2.3. Evaluations

#### 2.3.1. Hand Motor Function

Motor function of both the affected and unaffected hands was assessed using the Wolf Motor Function Test (WMFT) [13] and the Action Research Arm Test (ARAT) [14]. The WMFT assesses motor function of the hand by probing 15 motor tasks relevant for daily life activities (putting the hand on the table, lifting a pencil, flipping cards, turning a key in a lock, etc.). The subtasks are scored by a scale ranging between zero and five points. The maximum achievable score is either 75 points (in patients with the ability to stand with assistance) or 70 points (in patients without the ability to stand independent). A higher score indicates better motor function. The ARAT is a 19-item score that is subdivided into four subtests (grasp, grip, pinch, and gross arm movement). The hand motor performance is rated on a four point (zero to three points) scale. The maximum score is 57 points. A higher score is associated with a better motor performance.

#### 2.3.2. Neurophysiological Evaluations

Neurophysiologic evaluations of the ipsilesional and the contralesional hemispheres comprised motor evoked potentials (MEP) recorded from the first dorsal interosseous (FDI) muscle, motor mapping of FDI muscle representation over the primary motor cortex, cortical silent period, and ipsilateral silent period. Transcranial magnetic stimulation was performed using a 70 mm figure-of-8 coil (Magstim, Dyfed, UK). Electromyographic activity was recorded using silver-silver-chloride electrodes positioned in a belly-tendon technique over the FDI muscle of the contralateral hand. The coil was placed tangentially in a posterior-anterior plane at a 45-degree angle from midline during all measures. For motor mapping a self-fabricated net cap was used. The cap consisted of one-by-one centimetre squares allowing an exact predefined positioning of the TMS coil over the scalp. TMS intensity was specified for each subject at baseline and kept constant during follow-up evaluations. LabChart and PowerLab software were used for data acquisition and analysis (AD-Instruments, Australia). Patients were seated in a comfortable chair during all experiments.

*(1) Hotspot and Resting Motor Threshold Detection*. Subjects were instructed to rest their hands on the lap throughout the experiments. First, the hotspots for both FDI muscles were identified. The hotspot was defined by the coil location where a single pulse of suprathreshold TMS consistently elicited the largest MEP from the contralateral FDI muscle. The resting motor thresholds (rMT) for both FDI muscles were identified. The rMT was defined as the lowest stimulator output intensity that elicited MEPs with peak-to-peak amplitudes of at least 50 *μ*V from the contralateral FDI muscle in at least five of ten trials.

*(2) Motor Evoked Potentials*. Subjects were instructed to rest their hands on their lap during the experiments. MEPs were sampled during TMS at 110% rMT. Peak-to-peak amplitudes of the MEPs evoked in the contralateral FDI muscle were measured. 20 MEPs were sampled and averaged for each hand and hemisphere.

*(3) Motor Map Area*. Subjects were instructed to rest their hands on their lap during the experiment. The motor map area (MMA) was sampled during TMS at 110% of rMT. The coil was positioned over the vertex (where stimulation did not produce a MEP) and was then moved in (i) anterior-lateral and (ii) posterior-lateral directions in 1 cm steps along the knots of the one-by-one centimetre squares of the net cap. At each position five stimuli were applied. The position was considered to be active, if at least two (of five) stimuli evoked MEP amplitudes of at least 0.2 mV in the contralateral FDI muscle. Based on this data, we calculated (1) MMA size (the number of active scalp positions), (2) MMA volume (the sum of MEP amplitudes of all active scalp positions), and (3) center of gravity (COG) (weighted average of all active scalp positions). The COG of each FDI muscle consists of two directions: *x*-direction (anterior-posterior) and *y*-direction (medial-lateral) with the center point lying over the vertex. The center of gravity for *x*-direction was calculated as (∑(MEP  size)*i* × (*X*  coordinate)*i*)/map  volume. The center of gravity for *y*-direction was calculated as (∑(MEP  size)*i* × (*Y*  coordinate)*i*)/map  volume [15].

*(4) Cortical Silent Period*. The CSP [16] duration was obtained from the voluntary contracted FDI muscle, after TMS at 130% of the rMT applied over the contralateral primary motor cortex. Participants were instructed to squeeze a force transducer in a lateral grasp between index finger and thumb, using 20–30% of maximum voluntary contraction. Accurate matching of contraction force was displayed on a computer screen and continuously monitored throughout the experiments. The CSP duration was measured from the beginning of the MEP until reoccurrence of any voluntary EMG activity. This is referred to as the absolute CSP and ends with a deflection of the EMG waveform [17]. 10 CSP recordings were obtained and averaged for each hand and participant.

*(5) Ipsilateral Silent Period*. The ISP duration was obtained from the voluntary contracted FDI muscle, after TMS at 150% of the rMT applied over the ipsilateral primary motor cortex. Patients were instructed to squeeze a force transducer in a lateral grasp between index finger and thumb as strong as they could (maximum voluntary contraction). Maximum voluntary contraction of the FDI muscle was displayed on a computer screen and continuously monitored throughout the experiments. ISP duration was measured from rectified EMG recordings. The ISP onset, which reflects the onset latency of transcallosal inhibition, was determined as the time point after TMS stimulus application when the first sign of significant decrease (>25%) in the mean rectified EMG activity level occurred. The ISP duration was measured from ISP onset to the first sign of recovery in the background EMG activity (ISP offset). 10 ISP recordings were obtained and averaged for each hand and participant.

*(6) Data Analysis*. The data was analyzed using SPSS Statistic 21 (International Business Machines Corporation Systems, Armonk, USA). The changes of motor function of the affected hand as well as the neurophysiological changes were calculated as differences to baseline (two weeks' evaluation, baseline; four weeks' evaluation, baseline; six weeks' evaluation, baseline). Intervention induced changes of motor function of the affected hand were evaluated by one way ANOVA (4 times). Pearson correlation-coefficients were calculated to assess possible relationships between hand motor function, neurophysiological values, and clinical outcome. For multiple comparison and analyses the Bonferroni correction was used.

## 3. Results

17 stroke patients were included. Four patients were lost during the six-week follow-up (see Figure 1).

### 3.1. Hand Motor Function

Patients demonstrated a broad spectrum of motor impairment of the affected hand, ranging from severe to mild as assessed by both hand motor function tests at baseline (see Table 1). The WMFT showed an improvement of motor function of the affected hand from baseline to the two-week (mean ± standard deviation: 2.9 ± 4.4 points), four-week (mean ± standard deviation: 5.4 ± 6.3 points), and six-week evaluations (mean ± standard deviation: 4.9 ± 4.6 points). Also the ARAT showed an improvement of motor function of the affected hand from baseline to the two-week (mean ± standard deviation: 3.1 ± 5.3 points), four-week (mean ± standard deviation: 4.1 ± 6.4 points), and six-week evaluations (mean ± standard deviation: 3.6 ± 6.2 points). The one way ANOVA showed significant effects of the factor time on WMFT (*F*_3,19_ = 8.1; *p* = 0.000) and on ARAT (*F*_3,19_ = 3.7; *p* = 0.021). Functional improvement of the affected hand ranged between −1 and 19 points for the WMFT and between 0 and 20 points for the ARAT.

### 3.2. Neurophysiological Evaluations

At baseline, MEPs from the ipsilesional hemisphere were evocable in only three patients (P2, P3, and P6). All these patients suffered from a mild or moderate motor deficit of the upper limb. In one patient (P1) with a mild hand motor impairment MEPs from the ipsilesional hemisphere were evocable at the two-week evaluation and in one patient (P14, with a severe motor impairment) at the six-week evaluation. MEPs from the contralesional hemisphere were evocable in all patients at all time points.

#### 3.2.1. Resting Motor Threshold

Two patients (P2, P3) showed a higher rMT within the contralesional hemisphere compared to the ipsilesional hemisphere at baseline. Both patients suffered from a mild motor impairment of the affected hand. All remaining patients showed a higher rMT within the ipsilesional hemisphere compared to the contralesional hemisphere. These data imply that a severe motor impairment of the affected hand is associated with an interhemispheric imbalance of cortical excitability towards the contralesional hemisphere, whereas a mild motor impairment may be associated with a shift of cortical excitability towards the ipsilesional hemisphere.

#### 3.2.2. Motor Evoked Potentials

**Figure 2:**
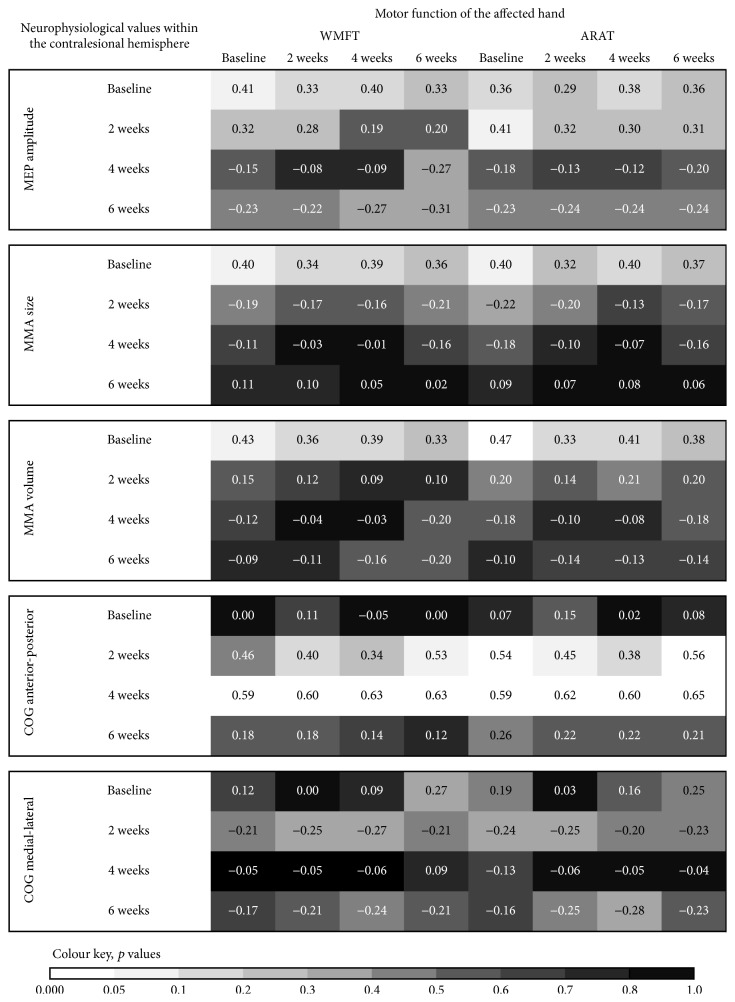
Correlation-coefficients between the neurophysiological values within the contralesional hemisphere and motor function of the affected hand.

**Figure 3:**
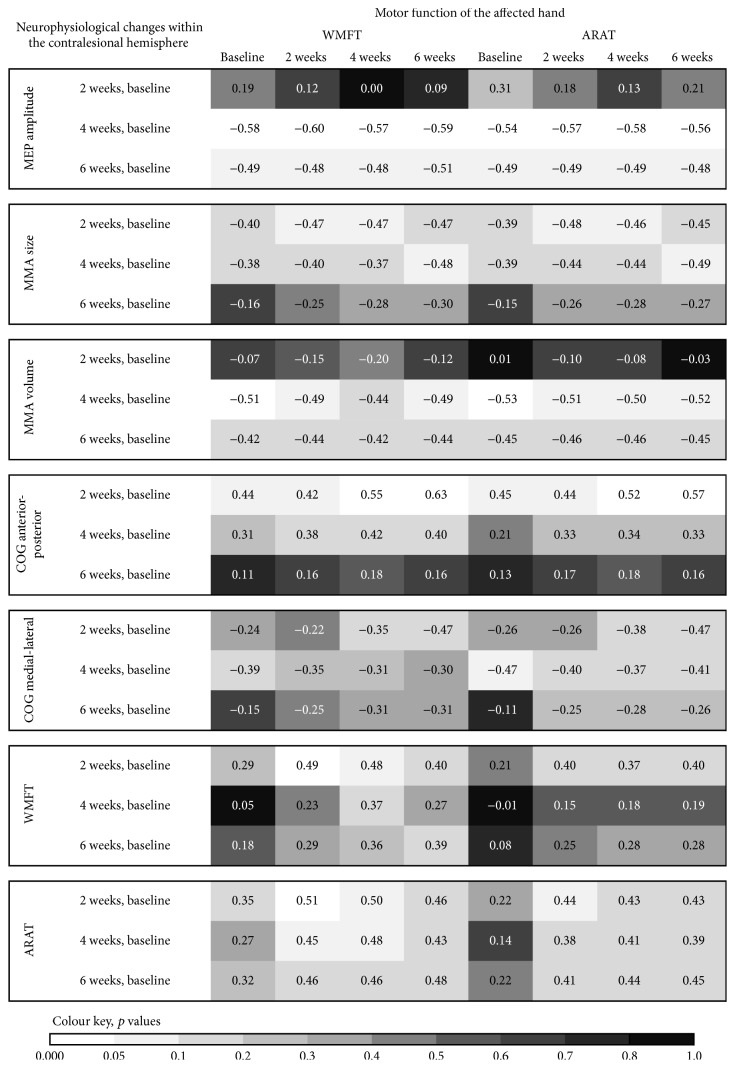
Correlation-coefficients between changes of neurophysiological values within the contralesional hemisphere and motor function of the affected hand.

The available data show greater MEP amplitudes after stimulation of the contralesional hemisphere, compared to stimulation of the ipsilesional hemisphere in all patients at baseline. The baseline data show positive correlations between MEP amplitudes within the contralesional hemisphere and motor function of the affected hand (see Figure 2). A low cortical excitability was associated with a poor motor function of the affected hand. The analysis of the long-term-data showed an increase as well as a decrease of MEP amplitudes elicited from the contralesional hemisphere over the following weeks. The changes ranged between −0.71 and +0.71 mV. Interestingly, the changes of MEP amplitudes elicited from the contralesional hemisphere correlated negatively with the motor function of the affected hand (see Figure 3). That is, patients with a mild to moderate deficit of motor function of the affected hand showed a decrease of cortical excitability two to six weeks from baseline. In contrast, patients with a severe motor deficit of the affected hand showed either an increase or a decrease of excitability of the contralesional primary motor cortex. In addition, a good neurological status at baseline was significantly correlated with a decrease of excitability within the contralesional primary motor cortex over time; for example, the decrease of MEP amplitudes from baseline to the follow-up examinations was positively correlated with NIHSS at baseline (*r* = 0.69, *p* = 0.004).

**Figure 4:**
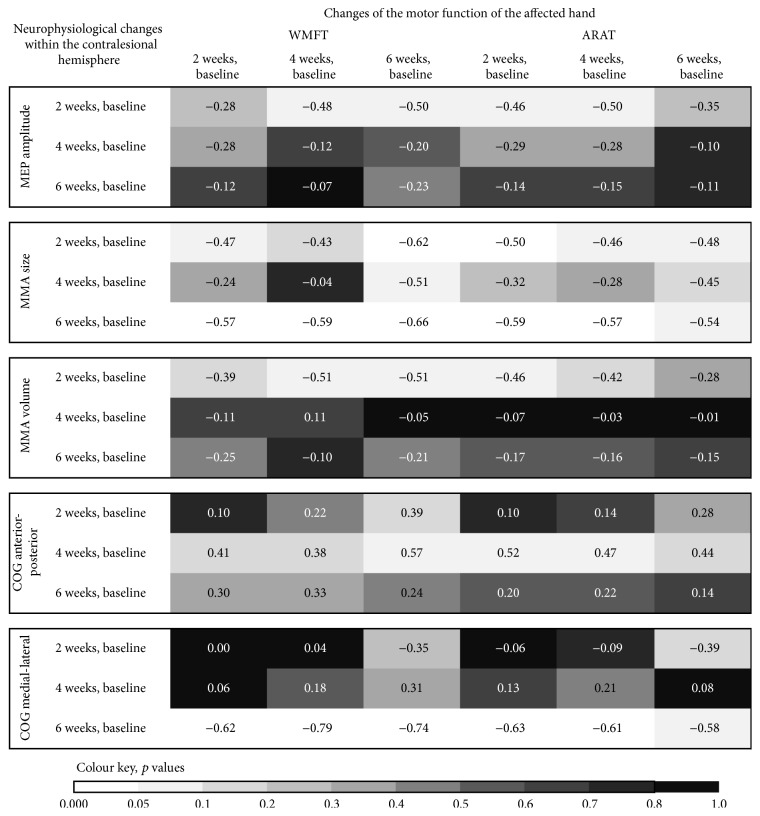
Correlation-coefficients between changes of neurophysiological values within the contralesional hemisphere and changes in motor function of the affected hand.

In addition, we found a number of nearly significant correlations between the changes of cortical excitability of the contralesional hemisphere and the changes of motor function of the affected hand (see Figure 4). The changes of motor performance of the affected hand were negatively correlated with the changes of excitability within the contralesional motor cortex. These results indicate that a decrease of cortical excitability of the contralesional hemisphere is associated with a good motor recovery of the affected hand after stroke. In contrast, an increase of contralesional cortical excitability was associated with a poor motor improvement. Only in three patients were we able to measure the MEP changes after stimulation of the ipsilesional hemisphere over time. In these, excitability of the ipsilesional motor cortex mainly decreased over the six weeks' follow-up. The changes ranged between −0.54 mV (decrease) and +0.15 mV (increase).

#### 3.2.3. Motor Map Area Size

At baseline MMA size was greater within the contralesional hemisphere compared to the ipsilesional hemisphere in all subjects. The correlation analyses showed positive correlations between MMA size within the contralesional hemisphere and motor function of the affected hand at baseline (see Figure 3). A good motor function of the affected hand was associated with a greater MMA size. The contralesional hemisphere showed an increase as well as a decrease of the MMA size over the follow-up period. The changes ranged between −14 (decrease) and +8 (increase) active points. Interestingly, after six weeks from baseline there was a decrease of the MMA size in most patients. In contrast, at the two-week follow-up, there was an increase of MMA size in the majority of patients. These data suggest that the direction of change of the MMA size varies depending on the time from stroke. The correlation analysis shows negative correlations between the changes of MMA size within the contralesional hemisphere and the motor function of the affected hand (see Figure 3). That is, an increase of MMA size within the contralesional hemisphere is associated with a more severe hand motor impairment. In contrast, a decrease of MMA size within the contralesional hemisphere was observed in patients with a mild or moderate motor impairment of the affected hand. The correlation analyses also showed negative correlations between the changes of the motor function of the affected hand and the changes of the MMA size within the contralesional hemisphere over the follow-up period (see Figure 4). Patients with a good motor improvement of the affected hand showed a decrease of MMA size within the contralesional hemisphere and those with a poor motor improvement of the affected hand an increase of contralesional MMA size.

The changes of MMA size within the ipsilesional hemisphere were measurable in only three patients, who suffered from a mild to moderate hand motor impairment. The data showed a decrease of the cortical hand motor representation size within the ipsilesional hemisphere in all subjects.

#### 3.2.4. Motor Map Area Volume

At baseline the MMA volume within the contralesional hemisphere was greater than that in the ipsilesional hemisphere in all patients. The baseline data show significant correlations between MMA volume and hand motor function (Figure 2). A severe motor impairment was associated with a small MMA volume within the contralesional hemisphere. During follow-up there was an increase as well as a decrease of the MMA volume within the contralesional hemisphere. The changes ranged between −4.4 and +5.8 mV. At the six-week follow-up evaluation there was a decrease of the MMA volume (compared to the baseline) in most patients. In contrast, at the two-week evaluation the MMA volume had increased in most patients. This indicates that the direction of change of the MMA volume varies in dependence of the time from stroke. The amount of hand motor impairment correlated negatively with the changes of the MMA volume within the contralesional hemisphere (Figure 3). Subjects with a severe hand motor impairment showed an increase of contralesional MMA volume over time, whereas subjects with a mild or moderate hand motor impairment showed a decrease. The changes of the contralesional MMA volume between baseline and the four-week evaluation correlated with the NIHSS at baseline (*r* = 0.56, *p* = 0.03). The changes of the contralesional MMA volume between baseline and the six-week evaluation correlated with the SIS (*r* = 0.63, *p* = 0.03) at baseline. These results imply that a good clinical status is associated with a decrease of contralesional MMA volume, while a poor clinical status is associated with an increase of contralesional MMA volume over the time from stroke. The changes of the motor function of the affected hand correlated negatively with the changes of the MMA volume within the contralesional hemisphere, most evident at the two-week follow-up evaluation (see Figure 4). This indicates that a reduction of MMA volume within the contralesional hemisphere may come along with a good motor recovery of the affected hand.

The changes of ipsilesional MMA volume were obtained in only three patients, all suffering from a mild to moderate hand motor impairment. In general, they showed a decrease of MMA volume over time. The changes ranged between −3.5 mV (decrease) and +0.2 mV (increase).

#### 3.2.5. Center of Gravity of the MMA

*(1) Anterior-Posterior Axis*. The data show several relevant correlations between the COG location along the anterior-posterior axis and motor impairment of the affected hand (Figure 2). Good motor function of the affected hand is associated with anterior location of COG within the contralesional hemisphere. Over the time of follow-up the COG of the MMA shifted along an anterior-posterior axis within the contralesional hemisphere. The changes ranged between +1,7 cm (anterior shift) and −3,7 cm (posterior shift). At week six from baseline there was a posterior shift of the MMA COG within the contralesional hemisphere in the vast majority of patients. This implies that a posterior shift of the contralesional MMA COG occurs during the later phase of recovery from stroke. We found positive correlations between motor function of the affected hand and the shift of MMA COG along the anterior-posterior axis within the contralesional hemisphere (Figure 3). A better motor function at baseline was indicative of an anterior shift of contralesional MMA COG. Our correlation analyses exhibited significant correlations between the NIHSS at baseline and an anterior shift of the contralesional MMA COG from baseline to the two-week evaluation (*r* = −0.62, *p* = 0.01) as well as the four-week evaluation (*r* = −0.49, *p* = 0.08). There was also a significant correlation between the MRS at baseline and the anterior shift of the contralesional MMA COG from baseline to the two-week evaluation (*r* = −0.57, *p* = 0.03). These results suggest an association between a good clinical status and an anterior shift of the contralesional MMA COG in the early phase of stroke recovery. In addition, motor improvement of the affected hand was positively correlated with the shift of the contralateral MMA COG along the anterior-posterior axis, which was most evident at the two-week follow-up (Figure 4). These results indicate that a good motor improvement is associated with an anterior shift of the MMA COG within the contralesional hemisphere within the early phase of motor recovery from stroke.

The anterior-posterior shift of the MMA COG within the ipsilesional hemisphere was obtained in only three patients. The changes ranged between −2.8 cm (posterior shift) and +0.6 cm (anterior shift).

*(2) Medial-Lateral Axis*. The follow-up tests demonstrated a lateral shift of MMA COG within the contralesional hemisphere over time in most patients. The changes ranged between 2.35 cm (lateral shift) and −1.45 cm (medial shift). The motor function of the affected hand was negatively correlated with the shift of the contralesional MMA COG along the medial-lateral axis (Figure 3). Patients with a severe motor impairment showed a lateral shift and, in contrast, those with a mild motor impairment a medial shift of the MMA COG over time. The change of motor function of the affected hand also correlated negatively with the shift of the contralesional COG along the medial-lateral axis (most evident at the two-week evaluation; Figure 4). This indicates that a lateral shift of the contralesional MMA COG was associated with a less well motor recovery of the affected hand.

The medial-lateral shift of the MMA COG within the ipsilesional hemisphere was obtained in only two patients. The changes ranged between −1.24 cm (medial shift) and +0.43 cm (lateral shift).

#### 3.2.6. Cortical Silent Period

The changes of the CSP after stimulation of the contralesional motor cortex ranged between −0.045 seconds (shortening) and +0.077 seconds (prolongation) over the follow-up period. Age of the included subjects correlated with the changes of the contralesional CSP duration from baseline to the four-week evaluation (*r* = 0.59, *p* = 0.02). Younger subjects demonstrated a decrease and elderly an increase of CSP duration over time. The time since stroke correlated significantly with changes of the contralesional CSP duration from baseline to the two-week follow-up (*r* = 0.74, *p* = 0.001), the four-week follow-up (*r* = 0.71, *p* = 0.003), and the six-week follow-up (*r* = 0.59, *p* = 0.03). Patients who were more than 40 days since stroke at baseline showed a marked increase of the CSP duration over time. This is in striking contrast to those patients, who were less than 40 days from stroke. It appears as if only the later phase after stroke is associated with an increase of CSP duration within the contralateral hemisphere. The SIS correlated significantly with the changes of the contralesional CSP duration between baseline and the six-week evaluation (*r* = −0.71, *p* = 0.009), suggesting that a severe sensory deficit is associated with an increase of the CSP duration over time, while a mild sensory deficit is associated with a decrease of CSP duration.

Follow-up measures of ipsilesional CSP were available in only three patients. The changes ranged between −0.042 seconds (shortening) and +0.31 seconds (prolongation).

#### 3.2.7. Ipsilateral Silent Period

The ISPs within the contralesional hemisphere were measurable in only three subjects. The follow-up changes ranged between −0.006 seconds (shortening) and +0.016 seconds (prolongation). The ISPs within the ipsilesional hemisphere were measurable in only two patients. The follow-up changes ranged between −0.009 seconds (shortening) and +0.005 seconds (prolongation).

## 4. Discussion

The objective of this study was to describe the plastic changes in cortical excitability and cortical hand motor representation during recovery from stroke and to relate potential changes to the clinical impairment and recovery of hand function. The overall aim was to establish potential electrophysiological surrogate markers that may help to judge upon outcome of hand motor function in an affected individual.

Indeed we found strong relationships between motor function/motor recovery of the affected hand and (i) the reduction of cortical excitability, (ii) the reduction in size and volume of cortical hand motor representation, and (iii) the medial and anterior shift of cortical hand motor representation within the contralesional hemisphere. Based on these data, both maladaptive and beneficial roles of the contralesional motor cortex on hand motor recovery after stroke may be discussed.

On one hand, our follow-up data suggest a strong association between the reduction of motor cortex excitability (MEP size, MMA size, and MMA volume) within the contralesional hemisphere and a more favorable hand motor recovery. This may be interpreted as a maladaptive role of the contralesional motor cortex for the process of hand motor recovery after stroke. Support to this notion comes from numerous FMRI studies that describe a significant relationship between enhanced motor related neural activity within the contralesional hemisphere and the amount of motor deficit of the stroke-affected hand, as well as a clear relationship between reduction of contralesional neural activity and a successful recovery of impaired motor function over time [6, 18, 19].

On the other hand, our data also show a strong relationship between hand motor function and motor recovery of the affected hand. Patients with a more severe impairment of upper limb motor function exhibit a less favorable recovery than those with mild disability and this is coupled with persistent enhancement of cortical excitability (MEP size, MMA size, and MMA volume) within the contralesional hemisphere over the follow-up-period. Thus, the severity of motor impairment is the predominant surrogate marker for a less favorable recovery and it may also trigger enhanced motor cortex excitability within the contralesional hemisphere. Possibly, the increased neural activity within motor areas of the contralesional hemisphere may be essential for hand motor performance in those patients with a more severe motor deficit after stroke. This theory receives support from a study demonstrating that an increase of MMA size within the contralesional hemisphere is associated with a good hand motor recovery in patients, who received a hand motor training [20]. In contrast, those patients not undergoing a targeted hand motor training show a less well recovery, coupled with a decrease of the contralesional MMA size [20].

At baseline testing we found significant relationships between cortical excitability within the contralesional hemisphere (MEP size, MMA size, and MMA volume) and motor function of the affected hand function: the lower the cortical excitability, the more severe the hand motor impairment. In contrast, patients with moderate to mild hand motor impairment demonstrated a greater cortical excitability.

Collectively, these results are in accordance with a recent review that investigated the evolution of cortical hand motor representation during the course of motor recovery after stroke [21]. Taken together these data indicate that severe hand motor impairment is associated with lower motor cortex excitability within the contralesional hemisphere, with the later increasing over the course of motor recovery. In contrast, less severe hand motor dysfunction is coupled with higher motor cortex excitability, with the later decreasing over the course of motor recovery.

**Figure 5:**
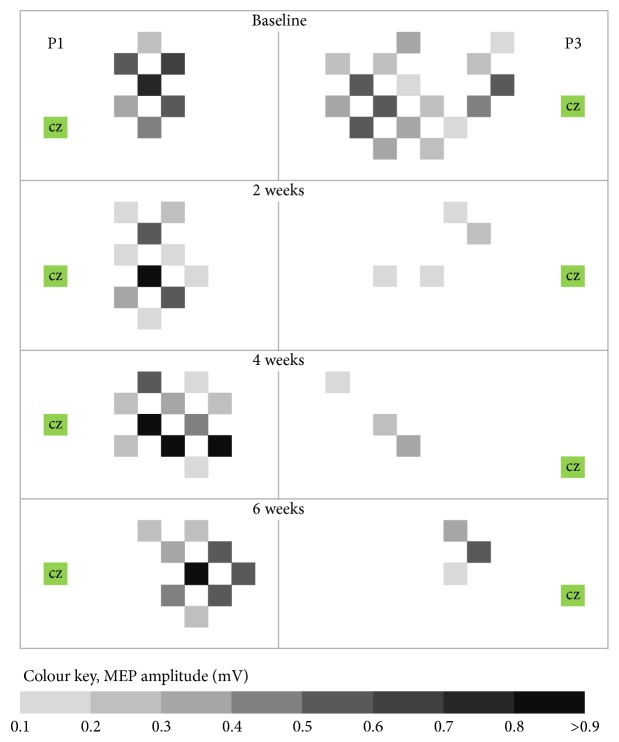
Hand motor representation area (MMA) within the contralesional hemisphere at baseline and its changes after six weeks in two representative patients. P3 shows a good motor recovery of the affected hand between baseline and the six-week evaluation (WMFT score increases by 16 points; ARAT score increases by 19 points). This was associated with a decrease in size and volume of contralesional MMA, as well as with an anterior-medial shift of hand motor representation. P1 shows a poor motor recovery of the affected hand at the six-week evaluation (WMFT score and ARAT score did not change). This was associated with an increase in size and volume of contralesional MMA and a posterior-lateral shift of hand motor representation. A = anterior direction; Cz = vertex; P = posterior direction.

Another interesting observation of the present study was that patients with a well preserved hand function and good motor recovery exhibited an anterior-medial shift of contralesional cortical hand motor representation. In contrast, a posterior-lateral shift of contralesional hand motor representation was found in those patients with a less favorable function and recovery of the affected hand (Figure 5). We can only speculate if an anterior-medial shift of contralesional hand motor representation represents positive plasticity, whereas a posterior-lateral shift is indicative of maladaptive plasticity in the process of recovery after stroke. A recent interventional study showed an association between a posterior shift of contralesional cortical hand motor representation and a more favorable outcome. Bobath therapy caused greater motor improvement coupled with a posterior shift of contralesional hand motor representation. In contrast, no additional therapy resulted in smaller motor improvements, coupled with no significant shift of cortical hand motor representation [20].

Our study protocol did not include neuronavigation based on brain imaging. Therefore we cannot comment on the exact anatomical landmarks of motor cortex plasticity within the contralesional hemisphere. Nevertheless, it appears as if a poor motor function and recovery were accompanied by an extension of contralesional hand motor representation towards the lateral surface of the frontal lobe and posterior towards the parietal lobe. In contrast, a favorable motor function and recovery were accompanied by a reduction of the cortical excitability within the aforementioned areas coupled with an extension towards premotor cortex and supplementary motor areas within the contralesional hemisphere.

In summary, we were able to show that recovery of motor function of the affected hand after stroke is accompanied by definite changes in excitability, size, volume, and location of hand motor representation within the contralesional primary motor cortex. These measures may be developed to serve as surrogate markers for motor outcome after stroke in the future.

  Acute stroke is within 2 weeks from symptom onset.
  Subacute stroke is 2 weeks to 3 months from symptom onset.
  Chronic stroke is more than 3 months from symptom onset.

## Figures and Tables

**(a):** 

Pat. Nr.	Sex	Age (years)	Time from stroke (days)	Stroke location	Stroke aetiology	Affected hem.	Dominant hem.	NIHSS (score)	MRS (score)	SIS (score)	MMS (score)	WMFT (score)		ARAT (score)	
												NAH	AH	NAH	AH
P1	m	66	15	CR	i	ri	le	0	1	na	na	70	66	57	57
P2	m	64	48	CR, GTS	h	le	le	0	2	6	29	70	62	57	53
P3	m	78	15	P	i	ri	le	0	2	12	28	70	60	57	57
P4	m	70	14	CR, GFS, pre-CG, post-CG	i	le	le	3	2	21	30	70	52	57	37
P5	f	76	22	BG	i	ri	le	1	3	16	28	70	47	57	28
P6	f	89	29	CR, pre-CG	i	le	le	2	4	7	27	70	40	57	20
P7	f	49	22	CR, GFI, GTS	i	ri	le	4	4	23	27	70	36	57	19
P8	f	44	22	CR, BG	i	ri	le	3	3	10	30	70	32	57	19
P9	m	76	50	CI, BG	i	le	le	4	3	6	29	70	28	57	19
P10	m	40	19	CR, BG	h	ri	le	5	4	26	28	70	18	57	0
P11	m	30	25	CI, GFI, GTS	i	le	le	8	4	25	28	70	16	57	0
P12	f	75	32	T, CI, CR	i	le	le	10	4	22	21	70	16	57	0
P13	m	80	30	BG	i	ri	le	na	na	na	na	70	16	57	0
P14	m	74	57	CR, post-CG	i	ri	le	3	3	19	29	70	16	57	0
P15	m	63	14	CR, BG, GFI, GFS, pre-CR, post-CG	i	ri	le	10	4	27	30	70	15	57	0
P16	w	63	16	CR, BG	h	ri	le	6	4	32	30	70	15	57	0
P17	f	76	94	CR, BG, pre-CG, post-CG	i	ri	le	na	na	na	na	70	15	57	0

AH = affected hand/affected hemisphere; ARAT = Action Research Arm Test; BG = basal ganglia; CI = capsule internal; CR = corona radiate; f = female; GFI = gyrus frontal inferior; GFS = gyrus frontal superior; GTS = gyrus temporal superior; h = haemorrhage; i = ischemia; le = left; m = male; MMS = Mini Mental Status Examination Score; MRS = Modified Rankin Scale; NAH = nonaffected hand/nonaffected hemisphere; NIHSS = National Institute of Health Stroke Scale; ri = right; P = pons cerebri; pre CG = precentral gyrus; post-CG = postcentral gyrus; SIS = Sensibility Impairment Score; T = thalamus; WMFT = Wolf Motor Function Test.

**(b):** 

Pat. Nr.	rMT (% of stimulator output)		MEP (mV)		MMA size (number of active sites)		MMA volume (mV)		COG anterior-posterior (cm)		COG medial-lateral (cm)		CSP (sec)		ISP (sec)	
	NAH	AH	NAH	AH	NAH	AH	NAH	AH	NAH	AH	NAH	AH	NAH	AH	NAH	AH
P1	64	>100	0.40	0.00	3	0	0.98	0.00	1.83	na	4.13	na	0.108	na	0.025	na
P2	85	75	0.84	0.23	9	5	4.35	1.02	2.18	−0.23	5.17	5.04	0.085	0.263	0.029	0.025
P3	68	57	1.40	0.76	12	9	7.81	3.71	2.70	4.26	3.59	5.48	0.159	0.183	0.033	0.034
P4	73	>100	0.53	0.00	8	0	2.53	0.00	1.95	na	5.27	na	0.187	na	na	na
P5	49	>100	1.15	0.00	12	0	5.11	0.00	1.10	na	3.41	na	0.107	na	na	na
P6	45	47	1.64	0.53	13	11	7.64	2.73	0.46	0.63	2.90	3.95	0.184	0.103	na	0.025
P7	75	>100	0.57	0.00	17	0	2.83	0.00	0.31	na	4.88	na	0.165	na	na	na
P8	66	>100	0.96	0.00	13	0	5.43	0.00	−0.15	na	5.55	na	0.158	na	na	na
P9	40	>100	0.49	0.00	5	0	1.54	0.00	3.01	na	2.96	na	0.156	na	na	na
P10	55	>100	1.11	0.00	7	0	4.30	0.00	1.03	na	4.01	na	0.160	na	na	na
P11	60	>100	0.18	0.00	2	0	0.35	0.00	3.16	na	5.32	na	0.174	na	na	na
P12	49	>100	0.71	0.00	7	0	3.50	0.00	1.38	na	2.89	na	0.183	na	na	na
P13	60	>100	0.35	0.00	5	0	1.15	0.00	0.70	na	4.98	na	0.154	na	na	na
P14	48	>100	0.21	0.00	1	0	0.21	0.00	0.00	na	4.24	na	0.091	na	na	na
P15	57	>100	0.65	0.00	11	0	3.66	0.00	3.68	na	4.04	na	0.197	na	na	na
P16	67	>100	0.28	0.00	4	0	0.82	0.00	1.45	na	2.98	na	0.163	na	na	na
P17	53	>100	1.37	0.00	13	0	6.29	0.00	−0.04	na	5.38	na	0.109	na	na	na

AH = affected hand/affected hemisphere; COG = center of gravity; CSP = cortical silent period; ISP = ipsilateral silent period; MEP = motor evoked potential; MMA = motor map area; mV = millivolt; na = not applicable/not available; NAH = nonaffected hand/nonaffected hemisphere; RMT = resting motor threshold.
